# A systematic review and individual bacterial species level meta-analysis of *in vitro* studies on the efficacy of ceftazidime/avibactam combined with other antimicrobials against carbapenem-resistant Gram-negative bacteria

**DOI:** 10.1093/jac/dkae451

**Published:** 2024-12-17

**Authors:** Getnet M Assefa, Jason A Roberts, Abdullah T Aslan, Solomon A Mohammed, Fekade B Sime

**Affiliations:** Centre for Clinical Research, Faculty of Medicine, The University of Queensland, Brisbane, QLD, Australia; Department of Pharmacy, College of Medicine and Health Sciences, Wollo University, Dessie, Ethiopia; Centre for Clinical Research, Faculty of Medicine, The University of Queensland, Brisbane, QLD, Australia; Pharmacy Department, Royal Brisbane and Women’s Hospital, Brisbane, QLD, Australia; Department of Intensive Care Medicine, Royal Brisbane and Women’s Hospital, Brisbane, QLD, Australia; Herston Infectious Disease Institute (HeIDI), Metro North Health, Brisbane, QLD, Australia; Division of Anaesthesiology Critical Care Emerging and Pain Medicine, Nimes University Hospital, University of Montpellier, Nimes, France; Centre for Clinical Research, Faculty of Medicine, The University of Queensland, Brisbane, QLD, Australia; Centre for Clinical Research, Faculty of Medicine, The University of Queensland, Brisbane, QLD, Australia; Department of Pharmacy, College of Medicine and Health Sciences, Wollo University, Dessie, Ethiopia; Centre for Clinical Research, Faculty of Medicine, The University of Queensland, Brisbane, QLD, Australia

## Abstract

**Background:**

Carbapenem-resistant Gram-negative bacteria (CR-GNB) develop resistance to many antimicrobials. To effectively manage infections caused by these organisms, novel agents and/or combinations of antimicrobials are required.

**Objectives:**

Evaluated the *in vitro* efficacy of ceftazidime/avibactam in combination with other antimicrobials against CR-GNB.

**Methods:**

PubMed, Web of Science, Embase and Scopus were searched. Study outcomes were quantified by counting the number of isolates exhibiting synergy, defined as a fractional inhibitory concentration index ≤ 0.5 for checkerboard and Etest, and a >2 log cfu/mL reduction for time-kill studies. The proportion of synergy was calculated as the ratio of isolates exhibiting synergy to the total number of isolates tested. These proportions were analysed using a random-effects model, following the Freeman–Tukey double-arcsine transformation.

**Results:**

Forty-five *in vitro* studies were included. A total of 734 isolates were tested, and 69.3% of them were resistant to ceftazidime/avibactam. The combination of ceftazidime/avibactam with aztreonam showed a high synergy rate against carbapenem-resistant *Klebsiella pneumoniae* (effect size, ES = 0.91–0.98) and *Escherichia coli* (ES = 0.75–1.00). Ceftazidime/avibactam also demonstrated a high synergy rate (ES = 1) in time-kill studies when combined with azithromycin, fosfomycin and gentamicin against *K. pneumoniae*. Compared to ceftazidime/avibactam alone, a higher bactericidal rate was reported when ceftazidime/avibactam was combined with other antimicrobials against carbapenem-resistant *K. pneumoniae* (57% versus 31%) and *E. coli* (93% versus 0%).

**Conclusions:**

Ceftazidime/avibactam frequently demonstrates synergistic bactericidal activity when combined with various antimicrobials against CR-GNB in *in vitro* tests. Further pre-clinical and clinical studies are warranted to validate the utility of ceftazidime/avibactam-based combination regimens for CR-GNB infections.

## Introduction

Carbapenem-resistant Gram-negative bacteria (CR-GNB) are a significant global public health concern because of their challenging resistance profiles, rapid spread within healthcare settings and substantial impact on morbidity and mortality. Consequently, the World Health Organization has categorized carbapenem-resistant Enterobacterales (CRE) and carbapenem-resistant *Acinetobacter baumannii* as critical priority pathogens and carbapenem-resistant *Pseudomonas aeruginosa* as high-priority pathogen. This designation underscores the urgent need for new therapeutic agents due to limited treatment options, high disease burden and increasing trends in antimicrobial resistance.^1^

Resistance to carbapenems is ascribed to various resistance mechanisms mainly determined by the type of bacterial species: diminished expression of porin to reduce uptake (e.g. OprD porin loss) and overproduction of efflux pumps (e.g. MexAB-OprM) in *P. aeruginosa*,^2,3^ altered expression of OmpK35/36 in *Klebsiella pneumoniae*^4^ and the expression of β-lactamase enzymes (i.e. carbapenemases) that can hydrolyze carbapenems in wide range of GNB.^5^

Carbapenemases are a common cause of carbapenem resistance in CR-GNB. *K. pneumoniae* carbapenemases (e.g. KPC), metallo-β-lactamases (MβLs) (e.g. NDM, VIM and IMP) and OXA-48-like carbapenemases (e.g. OXA-48 and OXA-181) represent the most clinically relevant carbapenemases encountered in GNB.^6^ To counteract the actions of these enzymes, novel β-lactam/β-lactamase inhibitors combinations such as ceftazidime/avibactam, meropenem/vaborbactam and imipenem/cilastatin/relebactam are now available and licenced for clinical use.

Ceftazidime/avibactam has activity against Class A and OXA-48-like carbapenemases, but not against MβLs.^7^ However, CRE isolates may develop resistance to ceftazidime/avibactam frequently due to the mutations in the Ω-loop region of the *bla_KPC_* gene and alterations of ompK35/36 porins.^8–10^ The risk of development of resistance to ceftazidime/avibactam during or following treatment is observed in up to 10% of cases, which can be identified as early as 10 days after treatment initiation.^11,12^ Consequently, relying solely on the clinical use of ceftazidime/avibactam might lead to the emergence of resistant isolates and subsequent treatment failure.

A strategically selected synergistic combination of ceftazidime/avibactam with other antimicrobials may help to mitigate the risk of resistance selection counteracting both inherent resistances mediated by MβL producers and acquired resistance by KPC producers.^10,13^ However, combination regimens are not without limitation; they may be associated with a higher risk of adverse effects, increased treatment cost and risk of resistance against concomitant antimicrobials.^14^ Therefore, selecting appropriate drug combinations whereby the potential benefit outweighs the risks poses a significant clinical challenge; particularly against GNB exhibiting high-level resistance to carbapenems. This is further complicated by the fact that it is ethically difficult, if not impossible, to conduct a clinical study that aims to identify synergistic antimicrobial combinations of high benefit. Thus, *in vitro* studies are required to evaluate the synergistic effects of drugs before clinical testing, and they can be invaluable tools to support clinical translation.^15^ A meta-analysis of observational studies has demonstrated a significant association between synergy-guided antimicrobial combination therapy against multidrug-resistant GNB and improved patient survival rates.^16^

Numerous *in vitro* studies have reported the efficacy of ceftazidime/avibactam in combination with other antimicrobials and evaluated their synergistic effects, however, to date, there has been no study that systematically pooled the *in vitro* synergistic activities of ceftazidime/avibactam combinations. Aslan *et al.*^17^ conducted a scoping review to assess the efficacy of both ceftazidime/avibactam monotherapy and its combinations, however, they did not perform meta-analyses due to the heterogeneous nature of aggregate data available from the studies. Therefore, this systematic review aimed to evaluate the *in vitro* efficacy of ceftazidime/avibactam in combination with other antimicrobials against CR-GNB by collating individual bacterial species-level data stratified by the *in vitro* testing method to enable a robust meta-analysis.

## Methods

### Literature search and screening

This study followed PRISMA guidelines, and the protocol was registered on PROSPERO (CRD42023483522). Using the PICO method, a search strategy was developed (Tables S1 and S2, available as Supplementary data at *JAC* Online) for online databases (PubMed, Web of Science, Embase, Scopus), and bibliographies were reviewed. The search was limited to English language publications until 13 September 2023, and Covidence software was utilized for data management and deduplication. Title, abstract and full document review were independently conducted by two authors (G.M.A. and S.A.M.), with disagreements resolved through discussion with a third author (F.B.S.).

### Selection criteria

#### Inclusion criteria

This study included *in vitro* studies using traditional synergy testing methods (time-kill, checkerboard and Etest) to evaluate the efficacy of ceftazidime/avibactam combined with other antimicrobials against CR-GNB.

#### Exclusion criteria

Studies were excluded if they assessed the synergistic effect of the combination on non-CR-GNB, evaluated the efficacy of ceftazidime/avibactam alone, evaluated the synergistic effect of avibactam with other antimicrobials, did not specify bacterial species (e.g. referring to them only as groups like Enterobacterales), or were abstracts, review articles, editorials, commentaries, or case reports.

### Data extraction

Data were extracted from texts, tables and graphs. The data extracted included the author’s name, publication year, *in vitro* combination testing methods, name of antimicrobials used, bacteria species, type of carbapenemases, number of isolates tested, number of isolates that show the outcome (synergy, antagonism, bactericidal and regrowth), initial bacteria inoculum size, number of replicates in the experiments and susceptibility of ceftazidime/avibactam and companion antimicrobials.

### Quality assessment tool

The quality assessment tool was developed based on a prior study,^18^ and consisted of 12 items organized under three domains. The first domain evaluated whether the research question was clearly stated. The second domain focused on assessing the methods and material used in the included studies. The final domain examined whether the outcome of interest was clearly defined. A detailed description of each criterion within these domains is presented in Table S3. G.M.A. and S.A.M. appraised the article’s quality, resolving any differences with the third author, F.B.S.

### Outcome measurement

The primary outcome measure was pharmacodynamic interaction described by synergy, additive, indifference, or antagonism. For checkerboard and Etests, pharmacodynamic interactions were expressed by the fractional inhibitory concentration index (FICI), calculated as described previously.^19^ Synergy was defined as FICI ≤ 0.5: additive, 0.5 < FICI ≤ 1; indifference, 1 < FICI ≤ 4; and antagonism, FICI > 4.^20^ For time-kill tests, synergy and antagonism were defined as a ≥2-log cfu/mL reduction or increment in bacterial load, respectively, at 24 h compared to the more active monotherapy.^21^

The secondary outcomes for time-kill studies were the bactericidal and regrowth rates. The bactericidal rate was defined as a greater than 3-log cfu/mL reduction in bacterial load at 24 h compared to the initial inoculum, and the regrowth rate was defined as a greater than 2-log cfu/mL decrease from the initial inoculum followed by an increase of ≥1-log cfu/mL at two subsequent time points (12 and 24 h).^22^

### Data analysis

Due to the heterogeneous nature of the studies, a traditional meta-analysis extracting aggregate (summary) data from each study was not undertaken. Instead, the analysis was done by extracting data for each species of bacteria and specific antimicrobials combined with ceftazidime/avibactam and stratified by the method used for synergy testing. For studies that undertake a second test to confirm synergy (e.g. time-kill tests were undertaken on selected isolates following checkerboard tests in some studies), only the first test was included in the meta-analysis. The number of isolates that show the study outcomes (synergy, antagonism, bactericidal, regrowth) was counted as events, and the proportion of the events was calculated as a ratio of the event count to the total number of isolates tested. The meta-analysis of the proportions of each event was done for each testing method (time-kill, checkerboard, Etests) separately using the random effect model. Studies evaluating the combination of the same drug with ceftazidime/avibactam were pooled together in each testing method. The pooled estimate of the study outcomes was calculated after the Freeman–Tukey double-arcsine transformation to stabilize the variances due to the extreme magnitude (0% and 100%) of the outcomes. Effect size (ES) was used to classify the pooled estimate of the primary outcomes; ES = 0—absence of the outcome, ES ≤ 0.35—low, 0.35 < ES < 0.75—moderate, ES ≥ 0.75—high. Positive trends were reported for combination regimens showing no significant 95% CI.^18^ The Metaprop package for R was used for meta-analysis.

## Results

### Study selection

**Figure 1.**
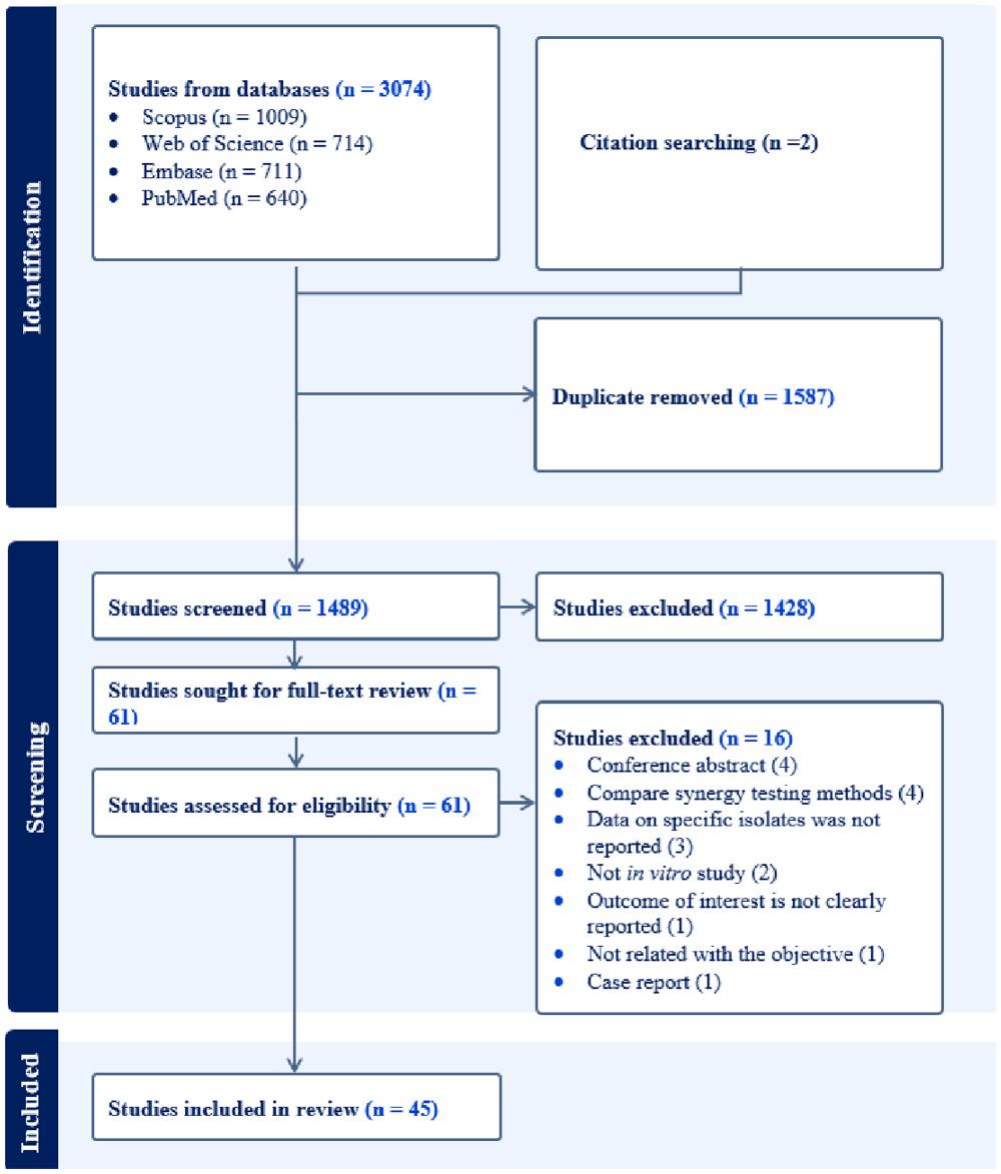
PRISMA flow chart showing the study selection process.

We initially identified 3076 papers through web-based searches and revision of bibliographies. However, 1587 were subsequently removed due to duplication. Out of the remaining 1489 studies that underwent title and abstract screening, 61 advanced to the full-text review stage, of which 16 were excluded. Ultimately, this review included 45 studies, as depicted in Figure 1.

### Quality assessments

From the 45 included studies, 12 studies reported all the quality assessment items, and others reported from 9 to 11 items of the quality assessment tool. The least reported items were the number of replicates of the experiment and the inclusion of quality control in the experiment reported by 21 and 23 studies, respectively (Table S4).

### Study characteristics

Twenty of the included studies were time-kill studies, 8 were based on checkerboard assays, and 17 used Etest methods.^23–67^ A total of 734 isolates [448 (61%) *K. pneumoniae*, 57 *P. aeruginosa*, 46 *A. baumannii* and 40 *Escherichia coli* from others] were tested in these studies, and 509 (69.3%) of them were resistant to ceftazidime/avibactam as presented in Table 1. A total of 140 combinations (61 by time-kill, 45 by Etest and 34 by checkerboard tests) were assessed for synergy.

**Table 1.** Characteristics of included studies

Author and year	Antimicrobials used in the combination	Bacterial species	Carbapenems resistance mechanisms	No of the isolates tested	CZA resistance profile	Synergy testing method(s)	Outcome measures reported
Almarzoky Abuhussain *et al*., 2018^23^	Amikacin	*K. pneumoniae*, *P. aeruginosa*	KPC, MexXY-OprM mutation	6	S	time-kill	tks, b
Avery and Nicolau, 2018^24^	Aztreonam	*E. coli*, *K. pneumoniae*	CMY, CTX-M-15, NDM-1, OXA, TEM, SHV, VIM, IMP-4, OKP-B-2, SFO-1	10	R	Etest	FICI
Avery *et al*., 2019^25^	Fosfomycin	*P. aeruginosa*	NR	16	R	Etest	FICI
Biag *et al*., 2019^26^	Aztreonam	*K. pneumoniae*, *E. coli*	NDM, TEM, OXA, SHV, CTX-M, CMY-2/FOX	8	R	time-kill	tks, b
Bianco *et al*., 2022a^27^	Aztreonam, cefiderocol	*K. pneumoniae*, *E. coli*, *Providencia rettgeri*	KPC, OXA-48, VIM, NDM	10	7R/3S	Etest	FICI
Bianco *et al*., 2022b^28^	Cefiderocol	*K. pneumoniae*	KPC	6	3R/3S	Etest	FICI
Boattini *et al*., 2023^29^	Cefiderocol, aztreonam	*K. pneumoniae*, *P. aeruginosa*	VIM, NDM	5	R	Etest	FICI
Borjan *et al*., 2020^30^	Polymyxin B	*K. pneumoniae*	KPC-3	3	1R/2S	time-kill	tks, b
Bulman *et al*., 2022^31^	Aztreonam	*K. pneumoniae*	KPC, CTX, OXA-48	2	1R/1S	time-kill	tks, b
Chandran *et al*., 2023^32^	Sulbactam	*A. baumannii*	NDM, VIM, OXA	6	R	time-kill	tks, b
Chen *et al*., 2021^33^	Amikacin	*K. pneumoniae*, *E. coli*, *A. baumannii*, *P. aeruginosa*, *E. cloacae*	NDM, KPC-2	21	R	checkerboard	FICI
Crémet *et al*., 2022^34^	Aztreonam	*S. maltophilia*	NR	40	R	Etest	FICI
Davido *et al*., 2023a^35^	Colistin, gentamicin, fosfomycin	*E. coli*	OXA-48, CTX-M-15	1	S	time-kill	tks, b
Davido *et al*., 2023b^36^	Colistin, gentamicin, fosfomycin	*K. pneumoniae*	KPC-2	1	S	time-kill	tks, b
Gaibani *et al*., 2019^37^	Meropenem/vaborbactam	*K. pneumoniae*	KPC, SHV, TEM	18	6R/12S	Etest	FICI
Gaibani *et al*., 2017^38^	Ertapenem, imipenem, meropenem, gentamycin, tigecycline, ciprofloxacin	*K. pneumoniae*	KPC	13	2R/11S	Etest	FICI
Gaudereto *et al*., 2019^39^	Meropenem	*A. baumannii*, *Serratia marcescens*	NDM, IMI, VIM, SIM, KPC, OXA	16	11R/5S	time-kill	tks
Huang *et al*., 2021^40^	Amikacin, gentamicin	*K. pneumoniae*	KPC-3	4	4S	time-kill	tks, b
Kuai *et al*., 2023^41^	Tigecycline, polymyxin B, amikacin, aztreonam, meropenem	*K. pneumoniae*, *E. coli*, *E. cloacae*	KPC-2, NDM	16	4R/12S	checkerboard	FICI
Lee *et al*., 2021^42^	Aztreonam	*P. aeruginosa*	VIM, IMP, OXA, PDC, VEB	5	R	time-kill	tks, b
Liang *et al*., 2023^43^	Polymyxin B	*A. baumannii*	OXA-like	21	R	checkerboard	FICI
Lu *et al*., 2022^44^	Aztreonam	*K. pneumoniae*, *E. coli*, *E. cloacae*, *Citrobacter freundii*, *Klebsiella oxytoca*	KPC, OXA, NDM, IMP	31	R	checkerboard	FICI
Ma *et al*., 2019^45^	Polymyxin B	*K. pneumonia*	KPC-2, OXA, SHV, TEM, CTX-M,	3	3S	time-kill	tks, b
Manning *et al*., 2018^46^	Polymyxin B, amikacin, tigecycline	*K. pneumoniae*	KPC, SHV, TEM, *ompK35*, *ompK36*	10	10S	time-kill	Tks, b
Mantzana *et al*., 2023^47^	Aztreonam	*K. pneumoniae*	KPC, MBL	100	R	checkerboard	FICI
Maraki *et al*., 2021^48^	Aztreonam	*K. pneumoniae*	NDM, VIM, KPC	40	R	Etest	FICI
Marshal *et al*., 2017^49^	Aztreonam	*K. pneumoniae*	NDM-1, CTX-M-15, TEM, SHV, DHA	1	R	time-kill	tks, b
Mataraci Kara *et al*., 2020b^50^	Colistin, tobramycin, doripenem, levofloxacin	*P. aeruginosa*	OXA, IMP, VIM	6	2R/4S	time-kill	tks, b
Mataraci Kara *et al*., 2020a^51^	Colistin, tobramycin, tigecycline, doripenem, levofloxacin	*K. pneumoniae*, *E. coli*, *E. cloacae*	OXA-48	7	6R/1S	time-kill	tks, b
Mikhail *et al*., 2019^52^	Amikacin, azithromycin, fosfomycin, meropenem, colistin	*K. pneumoniae*, *P. aeruginosa*	KPC-3, SHV, LAP-1, TEM-1, OXA, CTX-M-15,	4	4S	time-kill	tks, b
Monogue *et al*., 2016^53^	Aztreonam, tigecycline	*K. pneumoniae*	NDM, OXA-48, CTX-M	1	R	Etest	FICI
Montero *et al*., 2021^54^	Amikacin, aztreonam, meropenem, colistin	*P. aeruginosa*	OXA, VIM, GES, AmpC, OprD deficiency	21	7R/14S	time-kill	tks, b
Nath *et al*., 2018^55^	Amikacin, meropenem, polymyxin B	*K. pneumoniae*	KPC-2, KPC-3	4	4S	time-kill	tks, b
Ojdana *et al*., 2019^56^	Ertapenem, fosfomycin, tigecycline	*K. pneumoniae*	NDM, KPC, OXA-48	19	10R/9S	Etest	FICI
Palmbo *et al*., 2023^57^	Cefiderocol, sulbactam	*K. pneumoniae*, *A. baumannii*, *P. aeruginosa*	KPC, TEM, SHV, OXA, CMY, ADC-73, PDC-16, PER-1	6	4R/2S	Etest	FICI
Papa-Ezdra *et al*., 2023^58^	Aztreonam	*K. pneumoniae*, *E. cloacae*, *C. freundi*, *Enterobacter hormaechei*	NDM-1, KPC, CTX-M-15, OXA, AmpC, TEM, SHV, ACT,	21	12R/9S	Etest	FICI
Papalini *et al*., 2020^59^	Meropenem, tigecycline, fosfomycin	*K. pneumoniae*	KPC	3	1R/2S	checkerboard	FICI
Pragasam *et al*., 2019^60^	Aztreonam	*K. pneumonia*	NDM, OXA-48-like	12	3S/9R	checkerboard	FICI
Ranieri *et al*., 2023^61^	Aztreonam	*S. maltophilia*	NR	50	11R/39S	Etest	FICI
Romanelli *et al*., 2020^62^	Meropenem, imipenem, ertapenem, fosfomycin	*K. pneumoniae*	KPC	10	S	Etest	FICI
Shields *et al*., 2018^63^	Colistin	*K. pneumoniae*	KPC, TEM, SHV, CTX-M	16	16S	time-kill	tks, b
Taha *et al*., 2023^64^	Aztreonam	*K. pneumoniae*, *E. coli*	KPC, NDM, IMP, VIM, OXA-48, GES	100	96R/4S	Etest	FICI
Wang *et al*., 2021^65^	Amikacin, colistin, tigecycline	*K. pneumoniae*	KPC	30	S	checkerboard	FICI
Wenzler *et al*., 2017^66^	Aztreonam	*K. pneumoniae*, *P. aeruginosa*, *E. coli*, *C. freundi*, *E. cloacae*, *A. baumannii*	NDM, IMP, VIM, KPC, OXA	7	R	Etest	FICI
Wilhelm *et al*., 2023^67^	Aztreonam	*K. pneumonia*	KPC-2, NDM-1, CTX-M-15, SHV-187, TEM-181, OXA-1	4	R	time-kill	tks, b

b, bactericidal; CZA, ceftazidime/avibactam; FICI, fractional inhibitory concentration index; R, resistance; S, susceptible; tks, time-kill.

## Meta-analysis

### Synergy and antagonism rates

#### K. pneumoniae

The combination of ceftazidime/avibactam with aztreonam showed high synergy rates in all testing methods (ES = 0.91 in time-kill, 0.95 in checkerboard and 0.98 in Etests). The combination of ceftazidime/avibactam with azithromycin, fosfomycin and gentamicin shows a high synergy rate (ES = 1.00) in time-kill studies and with carbapenems in checkerboard (meropenem) and Etest studies (imipenem and meropenem). The combination of ceftazidime/avibactam with amikacin, colistin, meropenem and polymyxin B showed low antagonism rates in time-kill studies. No antagonism was reported in checkerboard and Etest studies against *K. pneumoniae* as shown in Table 2 and Figures S1 and S2.

**Table 2.** *In vitro* synergy and antagonism of ceftazidime/avibactam in combination with other antimicrobials against *K. pneumoniae* by test method

Tests method used	Antimicrobials combined with CZA	Number of studies	Number of the isolates tested	ES (95% CI)	Synergy rate	ES (95% CI)	Antagonism rate
Time-kill	Amikacin	5	23	0.43 [0.23; 0.66]	Moderate	0.09 [0.01; 0.28]	Low
	Azithromycin	1	2	1.00 [0.16; 1.00]	High	0.00 [0.00; 0.84]	No antagonism
	Aztreonam	4	11	0.91 [0.59; 1.00]	High	0.00 [0.00; 0.28]	No antagonism
	Colistin	4	23	0.35 [0.16; 0.57]	Low	0.35 [0.16; 0.57]	Low
	Doripenem	1	4	0.25 [0.01; 0.81]	Low	0.00 [0.00; 0.60]	No antagonism
	Fosfomycin	2	3	1.00 [0.29; 1.00]	High	0.00 [0.00; 0.71]	No antagonism
	Gentamicin	2	5	1.00 [0.48; 1.00]	High	0.00 [0.00; 0.52]	No antagonism
	Levofloxacin	1	4	0.50 [0.07; 0.93]	Moderate	0.00 [0.00; 0.60]	No antagonism
	Meropenem	2	6	0.50 [0.12; 0.88]	Moderate	0.33 [0.04; 0.78]	Low
	Polymyxin B	4	20	0.35 [0.15; 0.59]	Low	0.20 [0.06; 0.44]	Low
	Tigecycline	2	14	0.07 [0.00; 0.34]	Positive trend	0.00 [0.00; 0.23]	No antagonism
	Tobramycin	1	4	0.50 [0.07; 0.93]	Moderate	0.00 [0.00; 0.60]	No antagonism
Checkerboard	Amikacin	3	46	0.41 [0.27; 0.57]	Moderate	0.02 [0.00; 0.12]	Positive trend
	Aztreonam	4	131	0.95 [0.89; 0.98]	High	0.00 [0.00; 0.03]	No antagonism
	Colistin	1	30	0.00 [0.00; 0.12]	No synergy	0.00 [0.00; 0.12]	No antagonism
	Fosfomycin	1	3	0.00 [0.00; 0.71]	No synergy	0.02 [0.00; 0.71]	Positive trend
	Meropenem	2	15	1.00 [0.78; 1.00]	High	0.00 [0.00; 0.22]	No antagonism
	Polymyxin B	1	12	0.50 [0.21; 0.79]	Moderate	0.00 [0.00; 0.26]	No antagonism
	Tigecycline	3	45	0.02 [0.00; 0.12]	Positive trend	0.02 [0.00; 0.12]	Positive trend
Etest	Aztreonam	8	153	0.98 [0.94; 1.00]	High	0.01 [0.00; 0.05]	Positive trend
	Cefiderocol	3	11	0.55 [0.23; 0.83]	Moderate	0.00 [0.00; 0.28]	No antagonism
	Ciprofloxacin	1	13	0.00 [0.00; 0.25]	No synergy	0.00 [0.00; 0.25]	No antagonism
	Ertapenem	3	42	0.71 [0.55; 0.84]	Moderate	0.02 [0.00; 0.13]	Positive trend
	Fosfomycin	2	19	0.47 [0.24; 0.71]	Moderate	0.00 [0.00; 0.18]	No antagonism
	Gentamicin	1	13	0.00 [0.00; 0.25]	No synergy	0.00 [0.00; 0.25]	No antagonism
	Imipenem	2	23	1.00 [0.85; 1.00]	High	0.00 [0.00; 0.15]	No antagonism
	MER/VAB	1	18	0.72 [0.47; 0.90]	Moderate	0.00 [0.00; 0.19]	No antagonism
	Meropenem	2	23	1.00 [0.85; 1.00]	High	0.00 [0.00; 0.15]	No antagonism
	Sulbactam	1	2	0.50 [0.01; 0.99]	Moderate	0.00 [0.00; 0.84]	No antagonism
	Tigecycline	3	33	0.06 [0.01; 0.20]	Low	0.00 [0.00; 0.11]	No antagonism

CZA, ceftazidime/avibactam; ES, effect size, ES = 0—the absence of the outcome, ES ≤ 0.35—low, 0.35 < ES < 0.75—moderate, ES ≥ 0.75—high; MER/VAB, meropenem/vaborbactam.

#### E. coli

**Figure 2.**
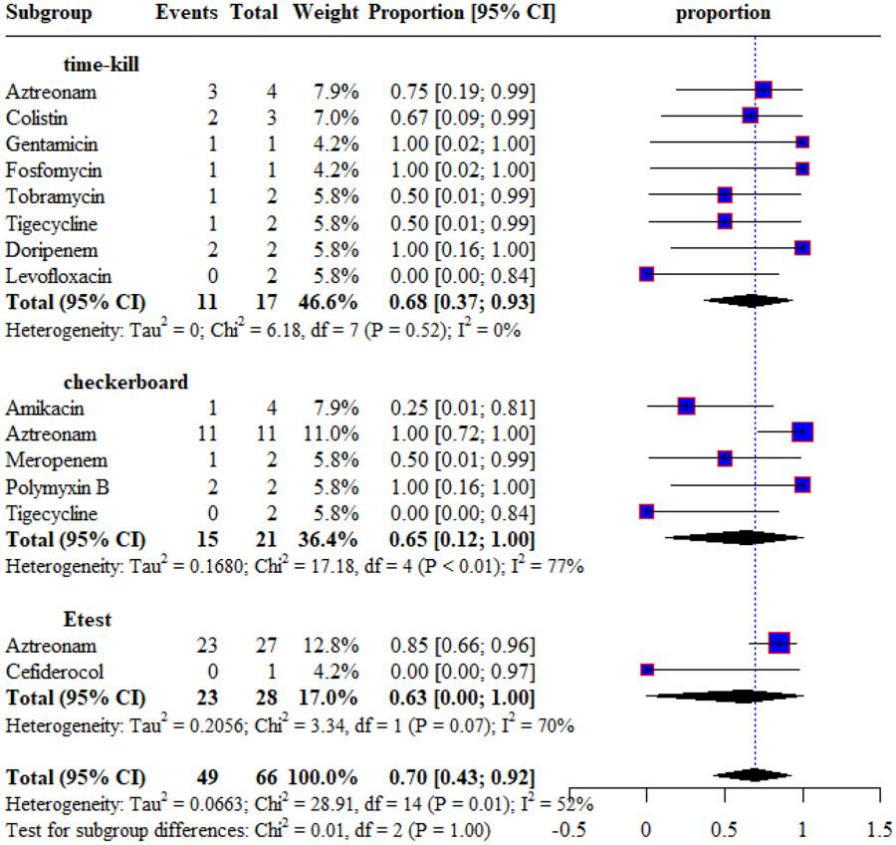
Synergistic effect of ceftazidime/avibactam with other antimicrobials against *E. coli*.

The synergistic effects of ceftazidime/avibactam with eight different antimicrobials against *E. coli* were assessed in three time-kill studies^26,35,51^ and the combinations showed moderate to high synergy rates, except with levofloxacin that did not show synergy. The synergistic effects of ceftazidime/avibactam combinations against *E. coli* were also reported by three checkerboard^33,41,44^ and four Etest studies.^25,27,64,66^ In all three testing methods, the combination of ceftazidime/avibactam with aztreonam showed a high synergy rate against *E. coli* (Figure 2). Antagonism was not reported in all testing methods against *E. coli*.

#### P. aeruginosa

**Figure 3.**
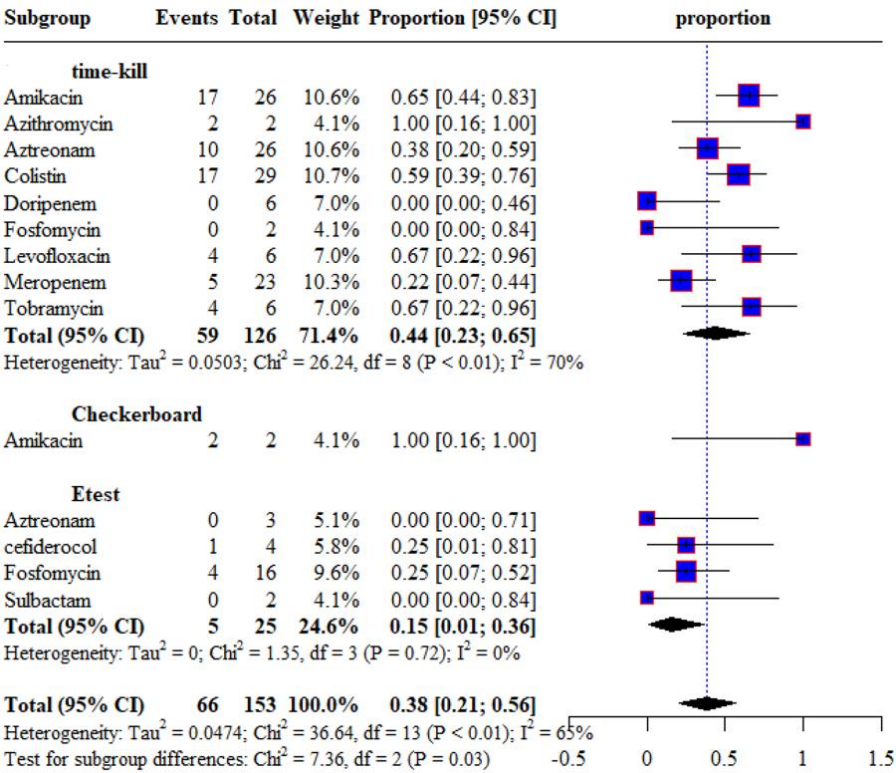
Synergistic effect of ceftazidime/avibactam with other antimicrobials against *P. aeruginosa*.

The synergistic effects of ceftazidime/avibactam with nine different antimicrobials against *P. aeruginosa* were reported by four time-kill studies.^42,50,52,54^ The synergy rate was high when ceftazidime/avibactam was combined with azithromycin and no synergy was noticed when combined with doripenem and fosfomycin. Only one study reports the synergistic activities of ceftazidime/avibactam combinations against *P. aeruginosa* using the checkerboard method.^33^ In the Etest study, the combination of ceftazidime/avibactam with fosfomycin showed a 25% synergy rate against *P. aeruginosa* as shown in Figure 3. Antagonism was reported only by one time-kill study when ceftazidime/avibactam was combined with meropenem against *P. aeruginosa* (19%).^54^

#### A. baumannii

The synergistic effect of ceftazidime/avibactam with meropenem and sulbactam was assessed by one time-kill study for each with a synergy rate of 3/6^39^ and 0/11^32^ isolates, respectively. A checkerboard microdilution study also reports the synergy of ceftazidime/avibactam with amikacin (one of five isolates)^33^ and polymyxin B (18 of 21 tested isolates).^43^ No antagonism was reported from either test method.

#### Stenotrophomonas maltophilia

The synergy of ceftazidime/avibactam with aztreonam against intrinsically carbapenem-resistant bacteria, *S. maltophilia*, was assessed using the Etest method, which showed a high synergy rate of 92.2% (83/90) with no antagonism.^34,61^

### Bactericidal and regrowth rate

In time-kill studies, the combination’s bactericidal effect against different species of bacteria was compared with that of ceftazidime/avibactam alone.

For *K. pneumoniae*, the bactericidal effects of ceftazidime/avibactam increased from 31% (95% CI, 16%–47%) when used alone to 57% (95% CI, 30%–82%) when combined with other antimicrobials. The combination exhibited a complete bactericidal effect when ceftazidime/avibactam was combined with azithromycin, fosfomycin and gentamicin. However, no bactericidal effect was reported when combined with tigecycline.

The bactericidal effects of ceftazidime/avibactam, both alone and in combination with other antimicrobials, were assessed against seven isolates of *E. coli*. Ceftazidime/avibactam did not exhibit a bactericidal effect on these isolates when used alone. However, in combination with other antimicrobials, it demonstrated a 93% (95% CI, 46–100%) bactericidal effect, including 75% with aztreonam and 100% with colistin, gentamicin and fosfomycin.

When comparing the bactericidal rates of ceftazidime/avibactam alone and in combination against 99 isolates of *P. aeruginosa*, the combination exhibited a lower bactericidal rate (54%, 95% CI: 26–81%) than ceftazidime/avibactam alone (57%, 95% CI, 45–68%). This difference was primarily attributed to the ceftazidime/avibactam–meropenem (22%) and ceftazidime/avibactam–colistin (43%) combinations, which showed a lower bactericidal effect compared to ceftazidime/avibactam alone (61%). However, the combinations of ceftazidime/avibactam with amikacin (74%), azithromycin (100%) and aztreonam (77%) demonstrated a higher bactericidal effect compared to ceftazidime/avibactam alone (61%, 0% and 54%, respectively).

The effect of the combined drugs and ceftazidime/avibactam alone on the regrowth rates of the bacteria was not reported in most studies or reported at different time points, making it impractical to pool and analyse this outcome.

## Discussion

In this systematic review and meta-analysis, we pooled individual bacteria species-level data from *in vitro* studies to assess and summarize synergistic interactions of ceftazidime/avibactam with other antimicrobials based on the species of bacteria, test methods used and individual antimicrobials combined with ceftazidime/avibactam.

The combination of ceftazidime/avibactam with aztreonam showed a synergistic effect against CR *K. pneumonia*, *E. coli* and *S. maltophilia*, irrespective of the test methods utilized, however, not against *P. aeruginosa.* This finding is consistent with Mauri *et al.*’s^68^ description of the ceftazidime/avibactam/aztreonam combination as a promising option against MβL-producing Enterobacterales. Aztreonam stands out as one of the β-lactams resistant to hydrolysis by MβLs, leading to its resurgence in clinical applications. Nonetheless, the utilization of aztreonam faces constraints due to the co-expression of extended-spectrum β-lactamases and AmpC-type β-lactamases with MβLs. The combination of aztreonam with avibactam may address this challenge with avibactam potentially inhibiting non-MβL β-lactamase enzymes thereby enhancing the spectrum of activity of the combination. The aztreonam–avibactam combination is not yet approved for clinical use^69^ as a result, clinicians use the ceftazidime/avibactam/aztreonam combination to treat serious infections caused by MβL producers.^70^ The Infectious Diseases Society of America guidance document also recommends the use of a ceftazidime/avibactam/aztreonam combination for the treatment of MβL-producing CRE infections.^9^ This innovative combination presents a promising solution for overcoming the limitations posed by enzyme-mediated resistance, allowing for broader clinical use of ceftazidime/avibactam and aztreonam.

A synergistic killing effect is often observed when drugs with distinct mechanisms or sites of action are combined.^71^ The combination of β-lactams and aminoglycosides, which act at different sites with distinct mechanisms, is widely used in clinical practice although the mortality benefit of these combinations remains controversial.^72,73^  *In vitro* studies encompassed in this review demonstrated a bactericidal synergistic effect when ceftazidime/avibactam is combined with aminoglycosides such as amikacin, gentamicin and tobramycin. This synergy is likely attributed to ceftazidime’s inhibition of cell wall synthesis, facilitating the entry of these aminoglycosides into the cytoplasm. Consequently, protein synthesis is hindered, intensifying the bactericidal effect of the combination. Moreover, aminoglycosides impede efflux pump activity and disrupt bacterial membrane permeability, further enhancing the bactericidal efficacy of ceftazidime/avibactam.^74,75^

Employing a combination of two β-lactams, each with distinct PBPs-binding patterns, may be useful for the treatment of severe infections. This approach enhances the bactericidal effect, potentially reducing the development of antimicrobial resistance.^76^ Studies using checkerboard and Etest methodologies have reported a high synergy rate when combining ceftazidime/avibactam with carbapenems that target PBP3 and PBP2, respectively, against *K. pneumoniae*, supporting the use of dual β-lactams. Additionally, ceftazidime-induced mutations in *bla*_KPC-3_ may reduce the enzymatic activity of KPC-3 due to conformational changes in the active site, thereby restoring susceptibility to carbapenems.^77^ However, the sustainability of this susceptibility is limited, and therefore, the clinical relevance of this finding remains unclear. Consequently, combining carbapenems with other agents is recommended for the treatment of infections caused by such strains.^78,79^

*In vitro* synergy tests serve as a base for the routine use of antimicrobial combinations in clinical practice.^16^ Since these tests follow different principles and measure different outcomes (inhibition versus killing), the presence of some discordance in the synergy rates is expected.^20^ For instance, checkerboard and Etest results reflect clinically used MIC values, while time-kill investigates the extent of bacterial killing over time, providing insights into the nature of the interaction. Time-kill tests are standardized and reproducible methods.^18^ However, both time-kill and checkerboard methods are time-consuming and labour-intensive. Therefore, identifying the test method that best predicts the *in vivo* efficacy of antimicrobial combinations is crucial for advancing the clinical extrapolation of *in vitro* results.

Furthermore, there is variability in how the outcome of interest is defined across studies. For instance, some studies consider antagonism as a FICI greater than 2, while others use it as greater than 4. Additionally, the regrowth rate is assessed at different time points in various studies, posing a challenge in integrating and comparing results across different studies. Standardizing the methodology for synergy testing, including precise definitions for the outcome of interest and implementing uniform reporting practices are crucial to facilitate the pooled data analysis from various studies.

The primary limitation of this study was the heterogeneous nature of data among the included studies. To reduce the effects of the heterogeneity on the meta-analysis results, we grouped the data extracted from the studies according to the species of bacteria used in the test (*K. pneumoniae*, *E. coli*, *P. aeruginosa*, *A. baumannii*), *in vitro* test methods used (time-kill, checkerboard and Etests) and specific antimicrobials combined with ceftazidime/avibactam. Secondly, the extensive diversity among strains within each bacterial species, coupled with varying resistance patterns, could potentially restrict the generalizability of the findings. Lastly, the regrowth rate of bacterial strains was either omitted in most studies or reported at disparate time intervals, rendering it unfeasible to aggregate and analyse this particular outcome.

In conclusion, this review highlights the notable *in vitro* synergy and bactericidal activity of ceftazidime/avibactam in combination with various antimicrobials against CR-GNB. However, caution is needed as *in vitro* results may not directly translate to clinical practice, given the inability to fully replicate *in vivo* conditions, including dynamic drug concentrations, bacterial inoculum, virulence and host immune responses at infection sites. To establish the efficacy of these combinations, further validation through pharmacokinetic/pharmacodynamic *in vitro* studies and multicentre randomized clinical trials is essential.

## Supplementary Material

dkae451_Supplementary_Data

## Supplementary data

Figures S1 and S2 and Tables S1–S4 are available as Supplementary data at *JAC* Online.
