# Supplementary material for: A systematic review and individual bacterial species level meta-analysis of in vitro studies on the efficacy of ceftazidime/avibactam combined with other antimicrobials against carbapenem-resistant Gram-negative bacteria
Source: J Antimicrob Chemother. 2024 Dec 17;80(2):334–46. doi: 10.1093/jac/dkae451 (PMC11787894; doi:10.1093/jac/dkae451)
Supplement: dkae451_Supplementary_Data [file dkae451_supplementary_data.docx]

**Supplementary material of “A systematic review and individual bacterial species level meta-analysis of *in vitro* studies on the efficacy of ceftazidime/avibactam combined with other antimicrobials against carbapenem-resistant Gram-negative bacteria”**

Getnet M Assefa, Jason A Roberts, Abdullah T Aslan, Solomon A Mohammed, Fekade B Sime

Table S1 Concepts developed for searching

| **Concept 1**  **(intervention)** | **Concept 2 (Synergy testing methods)** | **Concept 3**  **(population)** |
| --- | --- | --- |
| Ceftazidime AND (avibactam OR AVE1330A OR NXL104) | “In vitro” | “Carbapenem resistan*” |
|  | Checkerboard | Carbapenemase* |
|  | E-test | carbapenem-hydrolyzing |
|  | Etest | KPC |
|  | time-kill | NDM |
|  | Static | OXA-48 |
|  | dynamic | VIM |
|  | Synerg* | Imipenemase |
|  | Antagonist | “Extensive drug resistan*” |
|  | microdilution | “Pandrug-resistan*” |
|  | “Broth dilution” | “multi-drug resistan*” |
|  | “agar dilution” |  |
|  | “Hollow fiber” |  |
|  | combination |  |

Table S2. Search strategies used to retrieve articles from different databases (up to 13/09/2023)

| **Database** | **Search strategy** | **Results** |
| --- | --- | --- |
| PubMed | ("ceftazidime"[Title/Abstract] AND ("avibactam"[Title/Abstract] OR "AVE1330A"[Title/Abstract] OR "NXL104"[Title/Abstract]) AND ("In vitro"[Title/Abstract] OR "Checkerboard"[Title/Abstract] OR "E-test"[Title/Abstract] OR "Etest"[Title/Abstract] OR "time-kill"[Title/Abstract] OR "Static"[Title/Abstract] OR "dynamic"[Title/Abstract] OR "synerg*"[Title/Abstract] OR "Antagonist"[Title/Abstract] OR "microdilution"[Title/Abstract] OR "Broth dilution"[Title/Abstract] OR "agar dilution"[Title/Abstract] OR "Hollow fiber"[Title/Abstract] OR "combination"[Title/Abstract]) AND ("carbapenem resistan*"[Title/Abstract] OR "carbapenemase*"[Title/Abstract] OR "carbapenem-hydrolyzing"[Title/Abstract] OR "KPC"[Title/Abstract] OR "NDM"[Title/Abstract] OR "OXA-48"[Title/Abstract] OR "VIM"[Title/Abstract] OR "Imipenemase"[Title/Abstract] OR "extensive drug resistan*"[Title/Abstract] OR "pandrug resistan*"[Title/Abstract] OR "multi drug resistan*"[Title/Abstract])) AND (english[Filter]) | **640** |
| Web of Science | (TS=(Ceftazidime)) AND (((TS=(avibactam )) OR TS=(AVE1330A )) OR TS=(NXL104)) AND ((((((((((((((TS=(“In vitro”)) OR TS=(Checkerboard)) OR TS=(E-test)) OR TS=(Etest)) OR TS=(time-kill)) OR TS=(Static)) OR TS=(dynamic)) OR TS=(Synerg*)) OR TS=(Antagonist)) OR TS=(microdilution)) OR TS=(“Broth dilution”)) OR TS=(“agar dilution”)) OR TS=(“Hollow fiber”)) OR TS=(combination)) AND (((((((((((TS=(“Carbapenem resistan*”)) OR TS=(carbapenemase*)) OR TS=("carbapenem-hydrolyzing” )) OR TS=(KPC)) OR TS=(NDM)) OR TS=(OXA-48)) OR TS=(VIM)) OR TS=(Imipenemase)) OR TS=(“Extensive drug resistan*”)) OR TS=(“Pandrug-resistan*”)) OR TS=(“multi-drug resistan*”)) | **714** |
| Embase | (ceftazidime:ti,ab,kw) AND (avibactam:ti,ab,kw OR ave1330a:ti,ab,kw OR nxl104:ti,ab,kw) AND ('in vitro':ti,ab,kw OR checkerboard:ti,ab,kw OR 'e test':ti,ab,kw OR etest:ti,ab,kw OR 'time kill':ti,ab,kw OR static:ti,ab,kw OR dynamic:ti,ab,kw OR synerg*:ti,ab,kw OR antagonist:ti,ab,kw OR microdilution:ti,ab,kw OR 'broth dilution':ti,ab,kw OR 'agar dilution':ti,ab,kw OR 'hollow fiber':ti,ab,kw OR combination:ti,ab,kw) AND ('carbapenem resistance':ti,ab,kw OR carbapenemase:ti,ab,kw OR 'carbapenem hydrolyzing':ti,ab,kw OR kpc:ti,ab,kw OR ndm:ti,ab,kw OR 'oxa 48':ti,ab,kw OR vim:ti,ab,kw OR imipenemase:ti,ab,kw OR 'extensive drug resistance':ti,ab,kw OR 'pan drug resistance':ti,ab,kw OR 'multidrug resistance':ti,ab,kw) | 711 |
| Scopus | ((TITLE-ABS-KEY(Ceftazidime)) AND ((TITLE-ABS-KEY(avibactam) OR TITLE-ABS-KEY(AVE1330A) OR TITLE-ABS-KEY(NXL104)))) AND ((TITLE-ABS-KEY("In vitro") OR TITLE-ABS-KEY(Checkerboard) OR TITLE-ABS-KEY(E-test) OR TITLE-ABS-KEY(Etest) OR TITLE-ABS-KEY(time-kill) OR TITLE-ABS-KEY(Static) OR TITLE-ABS-KEY(dynamic) OR TITLE-ABS-KEY(Synerg*) OR TITLE-ABS-KEY(Antagonist) OR TITLE-ABS-KEY(microdilution) OR TITLE-ABS-KEY("Broth dilution") OR TITLE-ABS-KEY("agar dilution") OR TITLE-ABS-KEY("Hollow fiber") OR TITLE-ABS-KEY(combination))) AND ((TITLE-ABS-KEY("Carbapenem resistan*") OR TITLE-ABS-KEY(Carbapenemase*) OR TITLE-ABS-KEY(carbapenem-hydrolyzing) OR TITLE-ABS-KEY(KPC) OR TITLE-ABS-KEY(NDM) OR TITLE-ABS-KEY(OXA-48) OR TITLE-ABS-KEY(VIM) OR TITLE-ABS-KEY(Imipenemase) OR TITLE-ABS-KEY("Extensive drug resistan*") OR TITLE-ABS-KEY("Pandrug-resistan*") OR TITLE-ABS-KEY("multi-drug resistan*"))) AND ( LIMIT-TO ( LANGUAGE,"English" ) ) AND ( LIMIT-TO ( DOCTYPE,"cp" ) OR LIMIT-TO ( DOCTYPE,"ar" ) ) | **1009** |

Table S3 Quality Assessment Tool- customised from previous study (1).

| **Category of items** | **Questions** | **Score (No = 0, Yes = 1)** |
| --- | --- | --- |
| **Aim of the study** | 1. Was the research question of the study clearly stated? |  |
| **Materials and methods** | 1. Were the names and sources of the antimicrobials tested clearly stated? |  |
|  | 1. Were the concentrations of the antimicrobials used clearly stated? |  |
|  | 1. Were the species of the bacteria used in the study reported? |  |
|  | 1. Did the authors provide a detailed description of the method used to assess the research outcome? |  |
|  | 1. Was the antimicrobial susceptibility (MIC) determined using reference methods? |  |
|  | 1. Was quality control included in the experiment? |  |
|  | 1. Was growth control included in the experiment? |  |
|  | 1. Was the number of replicates of the experiment given? |  |
|  | 1. Was the study observation's duration and/or time point clearly described? |  |
|  | 1. Was the outcome analyzed with appropriate statistical methods? |  |
| **Results** | 1. Was the outcome of the interest (synergy, antagonism, bactericidal, regrowth) clearly stated? |  |

1. Scudeller L, Righi E, Chiamenti M, Bragantini D, Menchinelli G, Cattaneo P, et al. Systematic review and meta-analysis of in vitro efficacy of antibiotic combination therapy against carbapenem-resistant Gram-negative bacilli. Int J Antimicrob Agents. 2021;57(5):106344.

Table S4 Quality Assessment results of included studies

| S/No | study | testing method | Q1 | Q2 | Q3 | Q4 | Q5 | Q6 | Q7 | Q8 | Q9 | Q10 | Q11 | Q12 | TOTAL |
| --- | --- | --- | --- | --- | --- | --- | --- | --- | --- | --- | --- | --- | --- | --- | --- |
| 1 | Almarzoky Abuhussain et al., 2018 | Time-kill | 1 | 1 | 1 | 1 | 1 | 1 | 1 | 1 | 1 | 1 | 1 | 1 | 12 |
| 2 | Biag et al 2019 | Time-kill | 1 | 1 | 1 | 1 | 1 | 1 | 1 | 1 | 1 | 1 | 1 | 1 | 12 |
| 3 | Borjan et al 2020 | Time-kill | 1 | 1 | 1 | 1 | 1 | 1 | 1 | 1 | 1 | 1 | 1 | 1 | 12 |
| 4 | Chandran et al., 2023 | Time-kill | 1 | 1 | 1 | 1 | 1 | 1 | 1 | 1 | 1 | 1 | 1 | 1 | 12 |
| 5 | Kuai et al., 2023 | Checkerboard | 1 | 1 | 1 | 1 | 1 | 1 | 1 | 1 | 1 | 1 | 1 | 1 | 12 |
| 6 | Lee et al., 2021 | Time-kill | 1 | 1 | 1 | 1 | 1 | 1 | 1 | 1 | 1 | 1 | 1 | 1 | 12 |
| 7 | Ma et al., 2019 | Time-kill | 1 | 1 | 1 | 1 | 1 | 1 | 1 | 1 | 1 | 1 | 1 | 1 | 12 |
| 8 | Mataraci Kara et al., 2020a | Time-kill | 1 | 1 | 1 | 1 | 1 | 1 | 1 | 1 | 1 | 1 | 1 | 1 | 12 |
| 9 | Mataraci Kara et al., 2020b | Time-kill | 1 | 1 | 1 | 1 | 1 | 1 | 1 | 1 | 1 | 1 | 1 | 1 | 12 |
| 10 | Mikhail et al., 2019 | Time-kill | 1 | 1 | 1 | 1 | 1 | 1 | 1 | 1 | 1 | 1 | 1 | 1 | 12 |
| 11 | Montero et al., 2021 | Time-kill | 1 | 1 | 1 | 1 | 1 | 1 | 1 | 1 | 1 | 1 | 1 | 1 | 12 |
| 12 | Wilhelm et al., 2023 | Time-kill | 1 | 1 | 1 | 1 | 1 | 1 | 1 | 1 | 1 | 1 | 1 | 1 | 12 |
| 13 | Chen et al., 2021 | Checkerboard | 1 | 1 | 1 | 1 | 1 | 1 | 1 | 1 | 0 | 1 | 1 | 1 | 11 |
| 14 | Gaudereto et al., 2019 | Time-kill | 1 | 1 | 1 | 1 | 1 | 1 | 0 | 1 | 1 | 1 | 1 | 1 | 11 |
| 15 | Huang et al., 2021 | Time-kill | 1 | 1 | 1 | 1 | 1 | 1 | 1 | 1 | 0 | 1 | 1 | 1 | 11 |
| 16 | Liang et al., 2023 | Checkerboard | 1 | 1 | 1 | 1 | 1 | 1 | 1 | 1 | 0 | 1 | 1 | 1 | 11 |
| 17 | Lu et al., 2022 | Checkerboard | 1 | 1 | 1 | 1 | 1 | 1 | 1 | 1 | 0 | 1 | 1 | 1 | 11 |
| 18 | Manning et al., 2018 | Time-kill | 1 | 1 | 1 | 1 | 1 | 1 | 1 | 1 | 0 | 1 | 1 | 1 | 11 |
| 19 | Marshal et al., 2017 | Time-kill | 1 | 1 | 1 | 1 | 1 | 1 | 0 | 1 | 1 | 1 | 1 | 1 | 11 |
| 20 | Monogue et al., 2016 | Etest | 1 | 1 | 1 | 1 | 1 | 1 | 0 | 1 | 1 | 1 | 1 | 1 | 11 |
| 21 | Papalini et al., 2020 | Checkerboard | 1 | 1 | 1 | 1 | 1 | 1 | 0 | 1 | 1 | 1 | 1 | 1 | 11 |
| 22 | Davido et al., 2023a | Time-kill | 1 | 1 | 1 | 1 | 1 | 1 | 0 | 1 | 1 | 1 | 1 | 1 | 11 |
| 23 | Davido et al., 2023b | Time-kill | 1 | 1 | 1 | 1 | 1 | 1 | 0 | 1 | 1 | 1 | 1 | 1 | 11 |
| 24 | Avery and Nicolau, 2018 | Etest | 1 | 1 | 1 | 1 | 1 | 1 | 0 | 0 | 1 | 1 | 1 | 1 | 10 |
| 25 | Avery et al., 2019 | Etest | 1 | 1 | 1 | 1 | 1 | 1 | 1 | 0 | 0 | 1 | 1 | 1 | 10 |
| 26 | Bianco et al., 2022b | Etest | 1 | 1 | 1 | 1 | 1 | 1 | 1 | 0 | 0 | 1 | 1 | 1 | 10 |
| 27 | Boattini et al., 2023 | Etest | 1 | 1 | 1 | 1 | 1 | 1 | 1 | 0 | 0 | 1 | 1 | 1 | 10 |
| 28 | Mantzana et al., 2023 | Checkerboard | 1 | 1 | 1 | 1 | 1 | 1 | 0 | 1 | 0 | 1 | 1 | 1 | 10 |
| 29 | Maraki et al., 2021 | Etest | 1 | 1 | 1 | 1 | 1 | 1 | 0 | 0 | 1 | 1 | 1 | 1 | 10 |
| 30 | Ojdana et al., 2019 | Etest | 1 | 1 | 1 | 1 | 1 | 1 | 1 | 0 | 0 | 1 | 1 | 1 | 10 |
| 31 | Palmbo et al., 2023 | Etest | 1 | 1 | 1 | 1 | 1 | 1 | 0 | 0 | 1 | 1 | 1 | 1 | 10 |
| 32 | Papa-Ezdra et al., 2023 | Etest | 1 | 1 | 1 | 1 | 1 | 1 | 1 | 0 | 0 | 1 | 1 | 1 | 10 |
| 33 | Pragasam et al., 2019 | Checkerboard | 1 | 1 | 1 | 1 | 1 | 1 | 0 | 1 | 0 | 1 | 1 | 1 | 10 |
| 34 | Shields et al., 2018 | Time-kill | 1 | 1 | 1 | 1 | 1 | 1 | 0 | 1 | 0 | 1 | 1 | 1 | 10 |
| 35 | Wang et al 2021 | Checkerboard | 1 | 1 | 1 | 1 | 1 | 1 | 0 | 1 | 0 | 1 | 1 | 1 | 10 |
| 36 | Wenzler et al., 2017 | Etest | 1 | 1 | 1 | 1 | 1 | 1 | 1 | 0 | 0 | 1 | 1 | 1 | 10 |
| 37 | Bulman et al., 2022 | Time-kill | 1 | 1 | 1 | 1 | 1 | 1 | 0 | 1 | 0 | 1 | 1 | 1 | 10 |
| 38 | Nath et al., 2018 | Time-kill | 1 | 1 | 1 | 1 | 1 | 1 | 0 | 1 | 0 | 1 | 1 | 1 | 10 |
| 39 | Bianco et al., 2022a | Etest | 1 | 1 | 1 | 1 | 1 | 1 | 0 | 0 | 0 | 1 | 1 | 1 | 9 |
| 40 | Crémet et al., 2022 | Etest | 1 | 1 | 1 | 1 | 1 | 1 | 0 | 0 | 0 | 1 | 1 | 1 | 9 |
| 41 | Gaibani et al., 2017 | Etest | 1 | 1 | 1 | 1 | 1 | 1 | 0 | 0 | 0 | 1 | 1 | 1 | 9 |
| 42 | Gaibani et al., 2019 | Etest | 1 | 1 | 1 | 1 | 1 | 1 | 0 | 0 | 0 | 1 | 1 | 1 | 9 |
| 43 | Ranieri et al., 2023 | Etest | 1 | 1 | 1 | 1 | 1 | 1 | 0 | 0 | 0 | 1 | 1 | 1 | 9 |
| 44 | Romanelli et al., 2020 | Etest | 1 | 1 | 1 | 1 | 1 | 1 | 0 | 0 | 0 | 1 | 1 | 1 | 9 |
| 45 | Taha et al., 2023 | Etest | 1 | 1 | 1 | 1 | 1 | 1 | 0 | 0 | 0 | 1 | 1 | 1 | 9 |
|  |  |  | 45 | 45 | 45 | 45 | 45 | 45 | 23 | 29 | 21 | 45 | 45 | 45 | 478 |


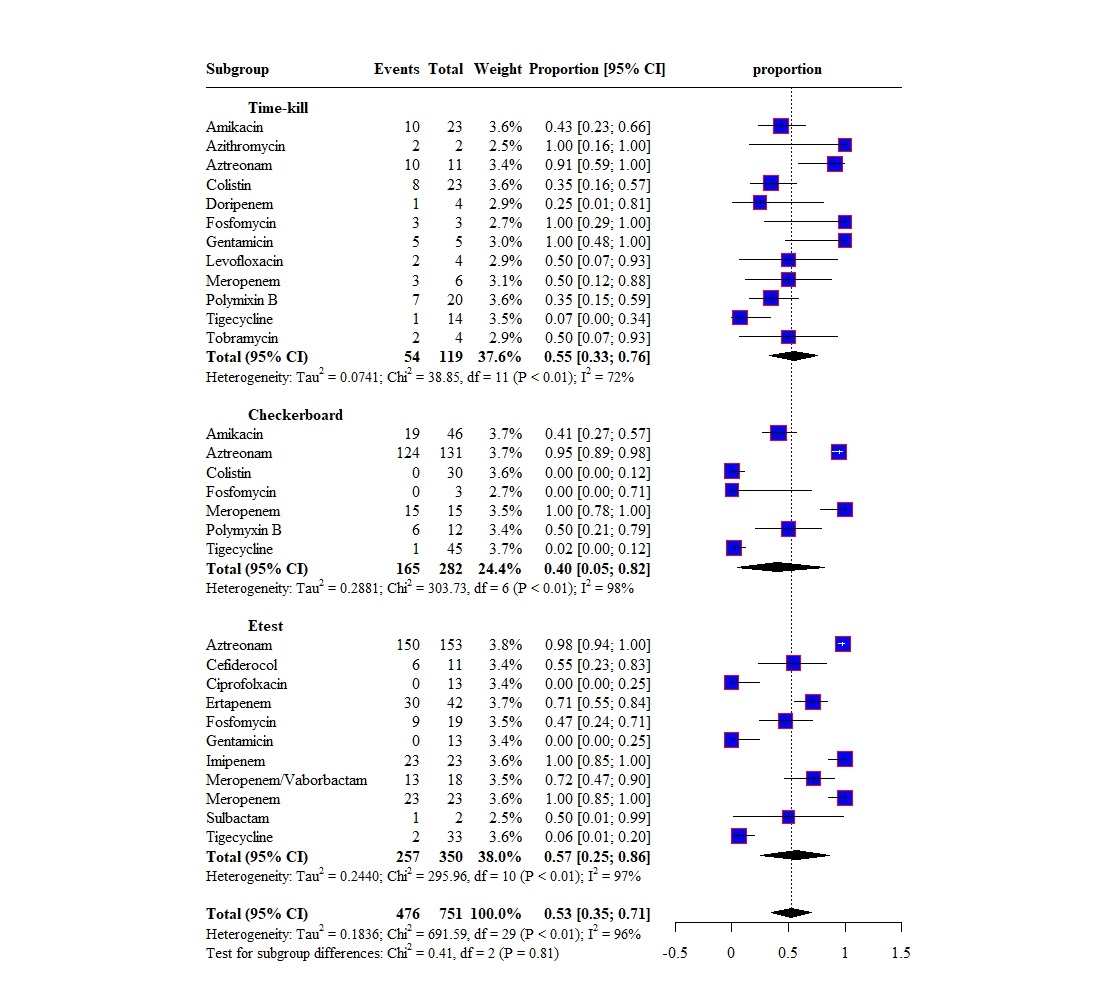


Fig S1. Synergistic effects of ceftazidime/avibactam in combination with other antimicrobials against K. pneumoniae


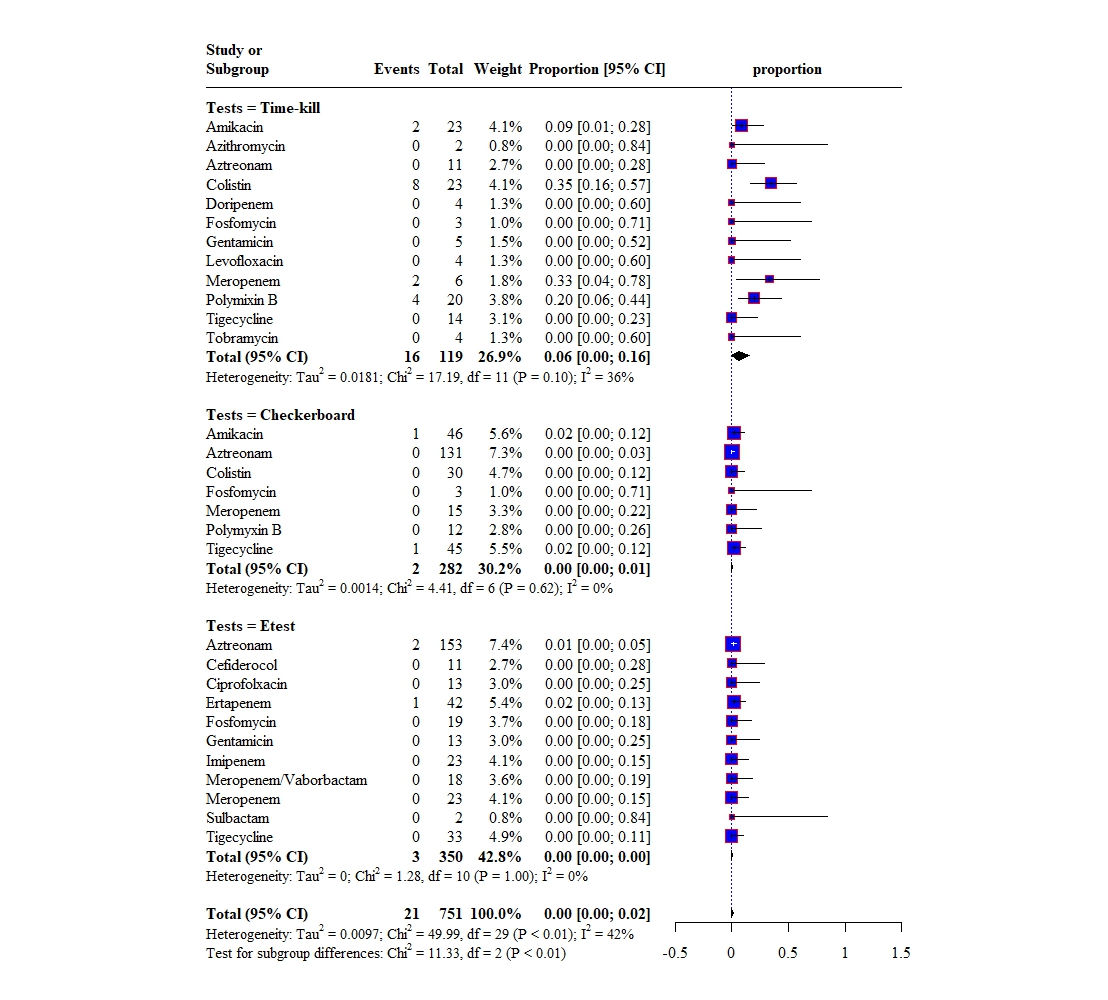


Fig S2. *In vitro* antagonism of ceftazidime/avibactam in combination with other antibiotics against *K. pneumoniae*
